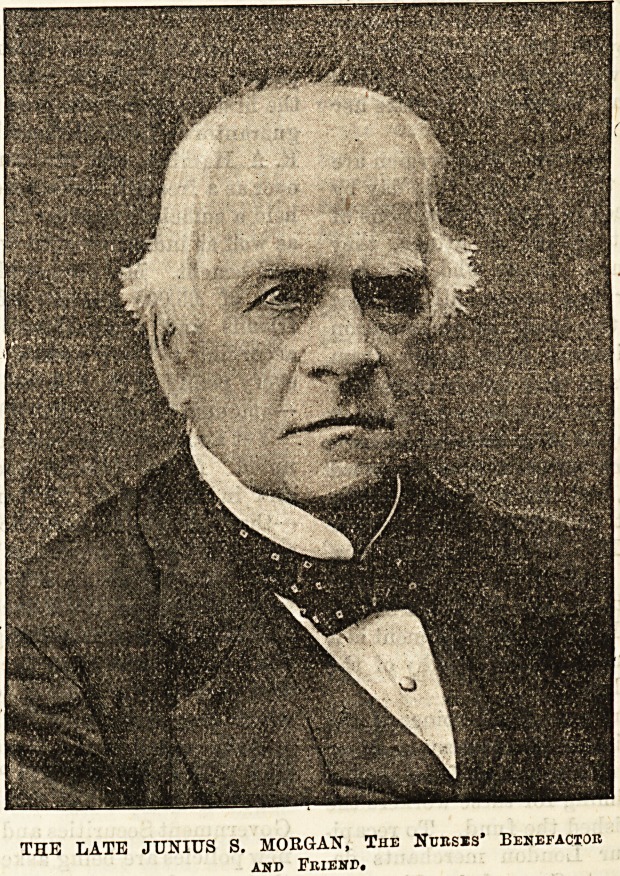# Extra Supplement—The Nursing Mirror

**Published:** 1890-06-28

**Authors:** 


Being the Extra Nursing Supplement of "The Hospital" Newspaper.

All Contributions for this Supplement should be addressed to the Editor, The Hospital, 140, Strand, London, W.C., and should have the word "Nursing" plainly written in left-hand top corner of the envelope.

### En Passant.

THE PRINCIPLE OF CO-OPERATION.—The minute for revising the payments of the private nursing staff in connection with the Hospital for Sick Children, Great Ormond Street, is an interesting document. It proves at once how generally it is now recognized as only just that a nurse should receive a fair share of her earnings. The fixed wage of the nurses is £25, uniform, and washing and travelling expenses. Then in their second year they also receive 10 per cent. on the sums paid for their services, which percentage rises yearly till it reaches 25 per cent.

WINCHESTER HOSPITAL.—The annual report of the Royal Hants County Hospital deals largely with nursing. The committee refer, with much satisfaction, to the very high repute which the hospital has attained for the skill and efficiency of its nursing staff. Applications are made from all quarters for the services of the private nurses attached to the hospital, and they almost invariably bring back the highest testimonials. The Committee, by way of reward and encouragement to their nursing staff, and having in view the profits accruing to the hospital from the private nursing system, resolved in June last—following the practice of other large hospitals—to afford some help towards providing sick pay and old-age allowances for nurses. "The National Pension Fund for Nurses" afforded an opportunity of effecting this object on the most advantageous terms. The Committee accordingly, after careful consideration, resolved to pay out of the private nursing fund, so long as the finances of the hospital would permit, one-half (limited not to exceed a certain amount) of the premiums payable by nurses who having been at least two years in the service of the hospital shall join the fund. Under the method of insurance adopted the nurse, if she retires from the profession, may (after having joined two years) withdraw all the contributions paid by her; and in the event of her death before the pension becomes due such contributions are returnable to her representatives and the moneys paid by the hospital on behalf of the nurse remain at the order and disposition of the hospital. With the aid thus afforded twenty-three members of the nursing staff joined the society. During the past year a most useful course of lectures on medical nursing was delivered by Dr. Harman to the nursing staff. It is intended that in future the instruction given to the nurses and probationers shall be more systematic and continuous. The Committee feel that their special thanks are due to Mrs. Suckling, the highly efficient matron and lady superintendent of nurses.

THE LATE JUNIUS S. MORGAN, The Nurses' Benefactor and Friend.

WOLVERHAMPTON NEWS.—The first annual meeting of the subscribers and friends of the Queen Victoria Nursing Institute at Wolverhampton was held on June 5th. The Home was chiefly started by the generosity of Mr. C. C. Smith, and has been most successful. The inauguration was on June 3rd, 1889, and in the middle of that month two nurses joined the institution. The first went out to a case at Llandudno on June 30th, and by the end of September there were seven on the staff all at work, and at the beginning of this year the number increased to thirteen, at which it still remains. Besides these, there are six probationers in training at various institutions. The nurses have attended 107 cases, and thus more than forty physicians and surgeons have had opportunities of judging of their work. Their reports, as well as those from the patients or their friends, have been almost without exception good. The lady superintendent, Miss Louisa Wenham, comes in for some well-earned praise, and the nurses are thanked for the alacrity with which they have taken disagreeable duties.

TRAINED MATRONS.—Mrs. Ashton Warner is circulating a leaflet amongst the ratepayers of the hamlet of Mile-end Old Town, stating how she is disqualified from acting as a guardian, to which post she had been elected. We hope someone will take up the work begun by Mrs. Ashton Warner when she gave notice of the following motion: "That in view of the opinion expressed by the Local Government Board in their letter dated February, 1883, viz., 'as to the matron, one of whose principal functions would be to superintend, subject to the directions of the medical superintendent, a large staff of female officers, it is of the highest importance that the person elected should have received thorough hospital training, and that her education and manner should be such as to inspire respect,' and in view of the fact that there is not one thoroughly trained nurse in the Mile-end Infirmary of 453 beds, from the matron downwards, steps be taken to replace the present matron of the infirmary (who has never been trained) by a lady who has gone through a complete course of training in one of the metropolitan hospitals, and that the present matron be asked to send in her resignation; that in taking this step in the interests of the poor sick patients, who must suffer severely from the untrained, and, therefore, unskilful nursing they receive, the Board desire that every consideration should be shown to the present matron for her long service." From 400 to 500 patients are tended in the infirmary by untrained nurses.

### The Fourth of July.

On the 4th of July the Princess of Wales will present the certificates of membership to the first thousand nurses who have joined the National Pension Fund. Of course there will be many who will ask contemptuously what the Princess has to do with nurses, and why the lady on whose shoulders fall so many of the active duties of royalty should take any interest in the affairs of an insurance company. But the Princess of Wales has been brought face to face with sickness and death, and knows the worth of trained and careful nursing. Furthermore, the establishment of the National Pension Fund for Nurses marks a further step in the development of woman. Day by day women are taking a more prominent part in national life. There is no longer any need to quote the proverb, *cherchez la femme;* satirists will tell you that nowadays woman is only too prone to come unsought and unsolicited to state her intentions and her views.

Satire apart, there can be no doubt that women are now an intellectual force in the land, and that day by day they are attacking and conquering new bastions of the citadels of labour. But, strong in attack, they are often weak in self-defence. Those who have had to do with masses of working women all say that it is difficult to teach them the lesson of co-operation and forethought. Their victories often profit them little; they become prisoners in their own strongholds. They cannot hold together, and they have nothing to fall back on. They are subject to certain delusions—for example, that the feminine system can be supported on tea and bread and butter, and that it is rather mean to try and provide for that dark time, when neither man nor woman can work. Now, the National Pension Fund for Nurses is the first attempt on a scale of any magnitude made by women to provide for sickness and old age. Looked at in this light, its establishment is a matter of no slight importance, and one worthy of the notice of the highest in the land.

The First Thousand Nurses are the heroines of the Pension Fund. We are doing no discourtesy to either the founder or the donors of the magnificent bonus fund in saying this, and claiming for these workers the distinction of having established the fund. To recapitulate an oft-told tale—four London merchants deposited with the Accountant-General in Chancery the sum of £20,000 as a guarantee to the policy-holders that their pensions would be paid, on the sole condition that a thousand policies should be taken out before the beginning of the present year. Now it is not too much to say that nurses, as a class, know nothing of the principles of life insurance. The tables of rates are an arithmetical mystery, and their idea of the amount of interest money should produce would seem excessive to Shylock. Not that they are greedy, but they are ignorant. Nor could many of them appreciate the care taken by the Council of the fund to secure that the investments should be beyond risk. Timid and well-meaning friends advised the savings bank or the old stocking, "and then you know exactly what you have, my dear;" and others who were neither friendly nor well-meaning sounded an alarmist note. We speak without malice in saying that the National Pension Fund has been the object of wilful misrepresentation. Let that be. We need not go out of our way to slay the slain, now that many more than a thousand nurses have found out that they do *not* lose all the premiums that they have paid if, one year, illness or accident renders it impossible for them to send the usual instalment; that the whole income of the bonus fund does *not* go to highly-paid officials; that if a nurse marries or dies before claiming her pension her premiums are *not* confiscated, and that other statements of similar character, once loudly sounded in their ears, are equally inaccurate. These thousand nurses had the brains to investigate the proposals of the Pension Fund, and the courage to come forward and invest their little savings in it, and it is they who have established a valuable institution, not only for their own benefit, but for that of the more timid sisters who, now that all semblance of risk had passed, came forward with their contributions. It is the first thousand who won the Bonus Fund, which the guarantors—Lord Rothschild, Mr. H. H. Gibbs, Mr. E. A. Hambro, and Mr. Junius Morgan—now handed over as a free gift, convinced that the nursing profession held a sufficient number of women, prudent and thrifty as well as industrious, to whom such a gift would be a real benefit. Mr. Junius Morgan added to his original gift of £5,000 another of the same amount, other friends and sympathisers followed with donations according to their means, till now the Bonus Fund—every penny of the interest of which goes to the nurses—amounts to nearly £40,000.

Yet another contribution to the Bonus Fund must be noted. A few months ago Mr. Junius Morgan died, and in memory of his beneficence the first thousand—that brave and energetic first thousand!—have resolved to form a fund that shall bear his name. They have been collecting actively among their friends and well-wishers, and the Princess of Wales has consented to receive purses containing contributions on July 4th. We do not doubt that the offerings will amount to a sum that will do honour to the grateful thought from which they sprung.

With regard to the Pension Fund itself, is it necessary to say anything now? Most of our readers know that the Council has invested upwards of £65,000 in Government Securities and others equally safe, and that new policies are being asked for every day. They know that many hospitals and nursing institutions have affiliated themselves to the Fund, desiring that their nurses should share in its benefits. Its career of well-doing and prosperity is thoroughly established, and it needs no advertisement now. But we are proud to congratulate the thousand nurses who have so largely contributed to that prosperity, and we congratulate on the position she has taken in the matter the Princess who so graciously gives her encouragement to the thrift and forethought they have so conspicuously displayed.

### ENTERTAINMENT AT MERCHANT TAYLORS' HALL.

The Master, Wardens, and Court of Assistants of the Merchant Taylors' Company have very generously placed their magnificent suite of Reception Rooms and the Hall at the disposal of the Council of the National Pension Fund for Nurses on Thursday evening, July 3rd. Every nurse who has joined the Pension Fund has been invited to meet the First Thousand, Mr.

W. H. Burns will preside, and Mr. Jonathan Hutchinson, F.R.S., President of the Royal College of Surgeons, has kindly promised to deliver an address. The Band of the Royal Engineers under the leadership of Herr Sowerthal, the Bandmaster, will be in attendance, and the attractions will include a Concert and other entertainments, which cannot fail to amuse and interest those who are fortunate enough to be present. In addition to the Council, the members, Governors, and donors of the £40,000, and several of the Patronesses and Vice-Presidents have been invited to meet the nurses. Refreshments will be supplied by Messrs. Ring and Brymer.

THE RECEPTION AT MARLBOROUGH HOUSE.

The arrangements for the ceremony at Marlborough House on Friday the 4th inst., are now completed. Each of the First Thousand Nurses who attends will be personally presented to the Princess, who will first receive the purses containing the money collected for the Junius S. Morgan Memorial Benevolent Fund, and then hand the certificates to each nurse. These certificates have been beautifully designed and etched by Miss Lillian C. Smythe, and will bear the autographic signature of the Princess of Wales as the President of the Fund. After the ceremony the Prince of Wales will address the nurses, and Mr. Walter Hayes Burns, the Chairman of the Council, will offer their Royal Highnesses the grateful acknowledgments of the nurses. The massed bands of the Guards will be in attendance, and should the day be fine this ceremony will no doubt prove one of the prettiest sights of the season. Nothing can exceed the kindness which their Royal Highnesses have displayed in connection with this occasion, and the personal interest that they have taken in the whole of the proceedings is as gratifying as it is remarkable. The event will be unique in the annals of nursing. Indeed, nothing so interesting has happened before in the history of the world in connection with workers amongst the sick.

### Little Babette.

"Little Babette!" is the half articulate cry of an old wounded veteran. He lies on the hard frozen ground helpless and starving. His uniform is tattered, and his uncovered limbs bleed from sores and blains. Little Babette comes hurrying up. One wonders how this little maid has found her way into the "Grand Army;" she is very little is Babette, small both of feature and stature, but her mien is modest and comely, and she has soft compassionate blue eyes, whose tender gaze seems to melt into what it beholds diffusing grace and mercy—and here, indeed, sight is but sense of suffering! The wounded soldier has not long to live. He expires in Babette's arms; but his death pangs are relieved by the maiden's ministrations, and a long unaccustomed smile moulds itself upon his lips as they stiffen into their final repose. Babette lays down the dead body reverently on the ground; there is no place nor time for burial here! Groans of the wounded and dying call to her on every side.

Is it not strange how, whilst all these strong men succumb to the hardships of the march, little Babette still struggles on uncomplaining. One would imagine that she never felt an ache! But surely her delicate little body must be vulnerable to the sharp arrows of cold and hunger and disease, at least as much as are the stronger frames of the weather-hardened warriors around her! They are wailing and despairing and dying; but she endures without a murmur! Hither and thither she flits about, a ministering angel, binding up this man's wounds, finding food for another, calming incipient madness in another, and so on; everywhere averting pain and soothing suffering.

She comes to the side of a young soldier, who cries to her: "For the love of God, kill me! Kill me!" His benumbed limbs refuse to be their own executioner. But Babette will not kill him—she restores the life in him. He feels the blood once more gurgling through his veins bringing warmth with it, and memory engenders in him thankful thoughts. How could he ever have wished to die? He must have been mad! And he calls down a blessing upon Babette for saving his life, that he may return to his young wife, who is watching and waiting for him at home.

In the distance may be heard gunshots and the roar of battle; but they soon cease. Oudinot with the vanguard has crossed over the bridges, and has repulsed a Russian attack on the opposite bank.

There is a stir in the camp, and the word is passed from mouth to mouth, "The Emperor!" All prepare themselves for his sight, and presently a faint cheer advances along the lines, "Vive L'Empereur!" and the great Napoleon passes by, stern visaged, with bent head and solemn brow, and glancing darkly in front, but neither to right nor left, of him. Babette looks up from her work of charity. Several officers approach the imperial general obeisantly with reports; but they are rebuked: "Why will you disturb my tranquillity? I desire to know no particulars! Why will you deprive me of my tranquillity?" And so the great Napoleon passes over the bridge, murmuring his habitual exclamation, "There is but one step from the sublime to the ridiculous!" and so he passes on and on into the ages!

Babette meanwhile pursues her self-imposed tasks. The soldiers continue their passage of the river—the greater number of them have reached the opposite banks. Suddenly the loud boom of a gun breaks upon the air, and immediately a long death line is swept through the serried ranks of the huddled-up multitude who yet await their turn to cross—another and another! Amidst shrieks of despair, a rush is made for the broader of the two bridges. Waggons, cannon, and human beings are all, ere long, literally piled upon the structure, when its supports give way, and the whole mass is precipitated into the dark, half-frozen waters of the stream beneath. High above the roar of artillery and the exultant "hurrahs" of the Cossacks resounds the piteous appealing clamour of that moment; but the crossing still goes on. There is another bridge, though it is very narrow. Meanwhile the Russian artillery rains death pitilessly.

Victor, who commands the French rear, holds his ground bravely; and by evening all his troops have crossed the bridge. The camp followers and retainers now may follow. It is Babette's turn! But she hears her name called, and she goes back. It is the young soldier before mentioned whose life she restored. Unable to walk, he had expected to have been taken over the bridge in a waggon; but the only bridge broad enough for a waggon has broken down, and he must now remain to his fate. He has a message and a memento of love for his wife at home—will Babette be the bearer of them? Of course she will! But he is weak, and his words come faltering and slow. Alas! Babette will never carry them home to his wife! When the French troops themselves have crossed over they set fire to the bridge, and even whilst Babette is drinking in the ill-fated man's message, the flames of the dry woodwork arise crackling in the air. The unarmed followers of the army, the sick, and the wounded are thus deliberately left to their fate; but their numbers are rapidly thinning—the Russian fire is continuous and deadly!

A ball strikes off one of the legs of the sick soldier. Babette dresses the wound: verily, it seems to rouse the animal of war in him, but it kills him. He dies, but his last words are not addressed to his wife, nor are they expressions of gratitude to Babette—his dying exclamation is the oft-repeated war-whoop, "Vive l'Empereur!"

Broken fragments of earth and ice are thrown into Babette's face; a cannon ball has just struck the ground in front of her, but she heeds not! Something seems to have recalled old memories; perhaps in this hour of hopelessness she permits herself a long forbidden dream—who knows?

But the hoofs of the Cossack horses ring out loud and clear on the hard surface of the neighbouring fields; and presently the swords and spears of their riders are no less busy. Their work is short! So is Babette's dream—unless, indeed, it be but the shadow of a grander one.

Thus dies a daughter of Peace, and the very name of her is soon forgotten: not so the man of War—a hundred generations hence the name of Napoleon will be living still! But in the End Peace will have her great reward, whilst War will perish!

### Amusements and Relaxation.

**N.B.—New Word Competition Commenced April 5th, 1890, ends June 28th, 1890.**

Proper names, abbreviations, foreign words, words of less than four letters, and repetitions are barred; plurals, and past and present participles of verbs, are allowed. Nuttall's *Standard* dictionary only to be used.

The word for dissection for this, the THIRTEENTH week of the quarter, being

"CERTIFICATE."

| Names. | June 19th. | Totals. | Names. | June 19th. | Totals. |
|---|---|---|---|---|---|
| Adeline | — | 149 | Agamemnon | 40 | 460 |
| Patience | 43 | 460 | G. E. J. C. | — | 34 |
| Lightowlers | — | 335 | S. E. A. | — | 24 |
| Canary | — | 86 | Jenny Wren | 33 | 416 |
| M. E. S. | — | 37 | Multum in parvo | — | 32 |
| Ambition | — | 36 | Ecila | — | 39 |
| M. W. | — | 217 | Weta | — | 259 |
| Esperance | — | 218 | Henri | — | 161 |
| Judy | — | 37 | Holland | 39 | 455 |
| Reldas | 38 | 455 | T. J. | — | 111 |
| Merenda | — | 29 | Nurse Isabel | — | 17 |
| Lucy Locket | — | 40 | Nurse Faith | — | 9 |
| Camellia | — | 30 | E. S. Z. | — | 10 |
| Qu'appelle | 31 | 429 | G. P. | — | 10 |
| Tinie | 43 | 463 | Stumps | — | 33 |
| Nurse Mildred | — | 190 | Bolton | — | 8 |
| Coralie | 35 | 446 | Jesmur | — | 53 |
| Gladys | — | 409 | Ran | — | 17 |

**Notice to Correspondents.**

*Second Quarterly Word Competition commences July 5th*, 1890.

N.B.—Each paper must be signed by the author with his or her real name and address. A *nom de plume* may be added if the writer does not desire to be referred to by us by his real name. In the case of all prize-winners, however, the real name and address will be published.

Competitors can enter for *all* quarterly competitions, but no competitor may take more than *one first prize* during the year.

### Vacancies.

The following vacancies are announced:

**Matrons.**

Halifax Fever Hospital; £50.

Sanatorium Clergy Daughters' School; £30.

**Housekeeper.**

Taunton and Somerset Hospital; £40

**Nurses.**

Barnstaple Nurses' Home; £25.

Barony Hospital, Glasgow (night); £24.

Belfast Nurses' Home.

Birmingham Eye Hospital; £25.

Blackheath Institute.

Braintree Workhouse; £22.

Brixton Institute.

Burton-on-Trent Institute; £25.

Cheltenham Hospital.

City Hospital, Newcastle-on-Tyne; £30.

Clapham Institute.

Edinburgh Longmore Hospital (night); £20.

Epsom Cottage Hospital; £20.

Glamorgan and Monmouthshire Infirmary; £24.

Greenwich Union; £20.

Halifax Union; £25.

Highbury Nurses' Institution.

Home for Nurses, Frome, £22.

Ipswich Nurses' Home; £22.

Leicester Fever Hospital; £25.

Levick Institution, Paris.

Middlesboro' Fever Hospital.

New Hospital for Women.

North-Western Fever Hospital; £36.

Scarborough Nurses' Institute.

Sheffield Nurses' Home.

South-Eastern Fever Hospital; £30.

Stockton-on-Tees Home.

St. Leonards, Shoreditch; £20.

Swansea Institute; £25.

Workhouse Infirmary Nursing Association.

Weston-super-Mare Institute.

**District Nurses.**

Birmingham Nursing Society.

Bolton, Lancashire; £25.

East London Nursing Society.

Hartlebury, Kidderminster.

Hulme District Nurses' Home; £22.

Manchester Sick Poor Institution.

**Probationers.**

Barrow-in-Furness Hospital; £12.
Brompton Hospital for Consumption.
Chelmsford Infirmary.
Clapham Institution.
Halifax Infirmary.
King's Lynn Hospital.
St. Helens Cottage Hospital.
Wrexham Infirmary.

### Appointments.

Beccles Hospital.—Miss M. E. MacDonnell has been appointed matron of this hospital in place of Mrs. Going, resigned. Miss Macdonald took her certificate at Edinburgh Royal Infirmary, where she nursed from November, 1887, till last February. We wish Miss MacDonnell every success in her new sphere.

Broad Oak Hospital.—Miss E. Goodburn was on May 30th appointed matron to this hospital at Hatfield, Essex. Miss Goodburn, trained at Liverpool Northern Hospital, in 1878, and was for three years matron of the Beverley Cottage Hospital. Miss Goodburn has had a large experience in private nursing, was matron at Ledbury, and lately had charge of the London and North-Western Railway Hospital at Crewe. She holds numerous and excellent testimonials and should give satisfaction at her new post.

Children's Shelter, Liverpool.—Miss Alice Conry has been appointed matron of the Children's Shelter, Liverpool. Miss Conry was trained at the Northern Hospital, Liverpool, and then acted as Sister—first in the children's wards, and latterly in the female wards of that hospital.

### Notes and Queries.

**Queries.**

(21) *New Tube.*—There is now a new vaginal tube for beginners, which is considered much safer for beginners to use when giving vaginal injections. Can anyone tell me where I can get it?—*Nurse Katie.*

(22) *Home Wanted.*—Can anyone tell me of a home in Hampshire for a man who is slightly imbecile? Friends can only pay small sum weekly.—*E. T.*

(23) *Cap.*—Will anyone tell me where I can obtain a "Sister Dora Cap?" Would buy new or second hand; or where a pattern could be obtained?—*M. E. W.*

**Answers.**

*The Fourth of July.—E. Murray.*—It is necessary that each nurse who intends to be present on the 4th should be present also on the 3rd. At the soirée the instructions will be given, which will secure the success of function before the Princess of Wales.

*Miss G. Clark and others.*—It is impossible to secure invitations for anyone to be present on the 4th save those policy holders of the first thousand who are personally going to receive their certificates.

*Nurse Smith.*—We only publish letters which are of general interest.

*A. Harvey.*—Great Yarmouth Hospital has 42 beds. The nursing staff consists of a matron and six nurses. You should consult the *Hospital Annual* on all these subjects. We make no charge for answering queries.

*Bookish.*—Others than hospital nurses are allowed to compete in the answers to the Examination Questions given in The Hospital, but they must state the fact and will not be eligible for the prizes.

*Constant Reader.*—The price of Dr. Dutton's pamphlet is Sixpence.

(15) *Home Wanted.*—Try 96, Upper Parliament Street, Liverpool, 7s. 6d. a-week; or, Midland Counties Home, Leamington.

*** Correspondents whose communications do not appear in the current number are requested to refer to the following week's issue.

## Figures and Tables

**Figure f1:**